# Temporal features of individual and collective self-referential processing: an event-related potential study

**DOI:** 10.7717/peerj.8917

**Published:** 2020-04-09

**Authors:** Cuihong Liu, Wenjie Li, Rong Wang, Yaohan Cai, Jie Chen

**Affiliations:** 1 School of Educational Science, Hunan Normal University, Changsha, Hunan, China; 2 Cognition and Human Behavior Key Laboratory of Hunan Province, Changsha, Hunan, China

**Keywords:** Self-referential processing, Individual, Collective, P2, N2, P3

## Abstract

**Background:**

Individual and collective self are two fundamental self-representations and are important to human experience. The present study aimed to investigate whether individual and collective self have essential difference in neural mechanism.

**Methods:**

Event-related potentials were recorded to explore the electrophysiological correlates of individual and collective self in a self-referential task in which participants were asked to evaluate whether trait adjectives were suitable to describe themselves (individual self-referential processing), a famous person (individual non-self-referential processing), Chinese (collective self-referential processing) or American (collective non-self-referential processing).

**Results:**

At the early stages, results showed that larger P2 and smaller N2 amplitudes were elicited by individual self-referential than by individual non-self-referential processing whereas no significant differences were observed between collective self-referential and collective non-self-referential processing at these stages. In addition, at the late P3 stage (350–600 ms), larger P3 amplitudes were also elicited by individual self-referential than by individual non-self-referential processing during 350–600 ms interval. However, the collective self-reference effect, indicated by the differences between collective self-referential and collective non-self-referential processing, did not appear until 450 ms and extended to 600 ms. Moreover, individual self-reference effect was more pronounced than collective self-reference effect in the 350–500 ms interval, whereas individual and collective self-reference effect had no significant difference in the 500–600 ms interval. These findings indicated that the time courses of neural activities were different in processing individual and collective self.

## Introduction

Self is an important issue in the field of psychology. Considerable studies demonstrated that self-relevant stimuli could be processed in a preferential manner, which can be dated back to so-called cocktail party effect (Conde, Gonçalves & Pinheiro, 2015; Liu et al., 2019; Rogers, Kuiper & Kirker, 1977; Su et al., 2010). For example, behavioral studies showed that people responded faster to one’s own face than to other faces even if arbitrary faces that are associated to self could also be processed preferentially (Ma & Han, 2010; Woźniak, Kourtis & Knoblich, 2018). Stimulus materials encoded in a self-referential way would be remembered better than other ways (Kesebir & Oishi, 2010; Symons & Johnson, 1997). More recently, event-related potentials, a high-temporal resolution technique recording neural activity with millisecond precision, have been widely used to investigate the time course of self-referential processing and its neural correlates. For example, electrophysiological studies found an obvious self-relevant effect on the early P2 component, which is a positive component peaking at the latency of around 200 ms after stimulus onset (Chen, Shui & Zhong, 2015; Liu et al., 2019). In addition, self-relevant effect could also occur on the N2 component, a negative deflection peaking between 200 and 350 ms after stimulus onset, with smaller N2 amplitudes induced by participants’ own than by other faces or handwritings (Chen et al., 2008; Keyes et al., 2010). Moreover, larger P3 amplitudes were also observed for self-relevant stimuli as compared to other stimuli (Chen et al., 2013; Conde, Gonçalves & Pinheiro, 2015; Tacikowski & Nowicka, 2010). For example, P300 amplitudes were found to be higher for self-face than for famous and unknown faces (Tacikowski & Nowicka, 2010). When evaluating whether trait adjectives were suitable to describe self or others, late positive component elicited in the self-referential condition was larger than that in other-referential conditions (Nowicka et al., 2018).

As described above, self-relevant stimuli receive preferential processing when comparing with other stimuli. However, these self-relevant stimuli such as participants’ names, faces (Tacikowski & Nowicka, 2010), hands (Su et al., 2010), handwritings (Chen et al., 2008) were confined to the individual level of self-concept. According to self-categorization theory, self-concept can be divided into individual and collective self, which emphasized one’s own uniqueness and relationship with others respectively (Ellemers, Spears & Doosje, 2002). It should be noted that collective self is also a fundamental self-representation (Brewer & Gardner, 1996; Ellemers, Spears & Doosje, 2002). In addition, some electrophysiological studies also demonstrated obvious collective self-reference effect (Fan et al., 2011; Yang, Liao & Huang, 2008; Zhao et al., 2009). For example, Tibetan students had better memory performances for trait adjectives encoded in reference to Tibetans than in reference to Han Chinese (Yang, Liao & Huang, 2008). In addition, P3 amplitudes elicited by collective self-referential stimuli (such as Alma mater name, self-national flag) were larger than that by collective non-self-referential stimuli (such as familiar and unfamiliar school names, familiar and unfamiliar flags), indicating that collective self-reference effect was obvious at late P3 stage (Zhao et al., 2009; Fan et al., 2011). Although collective self-reference effect exists, little is known whether individual and collective self engage attentional resources to a similar or different extent when comparing directly or whether individual and collective self-referential processing have similar or different temporal features.

As we have mentioned above, previous studies regarding self-referential processing mainly emphasized a single aspect of the self. It should be noted that self is a complex system and can be described from different dimensions (Marsh, Byrne & Shavelson, 1992; Zanden et al., 2015). For example, William James distinguished physical self from psychological self (James et al., 1890). In addition, self is continuous entity and involves not only present self but also past and future self (Luo et al., 2010). More recently, researchers have begun to investigate similarities and differences among different aspects of self in order to understand self comprehensively (Cygan et al., 2019; Liu et al., 2019; Zheng et al., 2018). For example, P300 amplitudes elicited by self-name (psychological self-related stimulus) and self-face (physiological self-related stimulus) were significantly correlated, indicating that self-name and self-face had very similar patterns of neural responses (Tacikowski & Nowicka, 2010). In addition, psychological (name) and physiological (voice) self-representations were demonstrated to have distinct neural responses in a auditory oddball task (Liu et al., 2019). Moreover, studies about temporal self demonstrated that present self was processed differently from past self (D’Argembeau et al., 2008; Yang et al., 2017). Taken together, these findings demonstrated that self could be understood comprehensively when comparing different components of self directly. Thus, our present study aimed to investigate the temporal features of individual and collective self-referential processing in a same study.

In addition, we used a self-referential task in which participants were presented with positive and negative personality trait adjectives and were required to make judgements on themselves (individual self), a famous person, Chinese (collective self) or American. Andy Lau, a famous Chinese superstar, was chosen as the control condition of individual self. It was considered that collective self emphasized the identity as the member of a social group and one’s nation was an important social group one belongs to (Yang & Huang, 2007; Zheng et al., 2018). Thus, “Chinese”, an important social identity of participants, was chosen to represent collective self, while “American”, which is equally familiar to participants, was chosen to represent non-collective self. Moreover, it was suggested that ERPs were suitable for investigating different cognitive processes on the basis of different components in the ERP waveform. Thus, we decided to use high-temporal resolution ERP technique to investigate whether individual and collective self have similar or different temporal features. Previous studies suggested that individual and collective self were both two important self-representations, but they were not equally important and meaningful (Gaertner et al., 2002, 2008, 2012). We predicted that individual and collective self-referential processing were different in terms of temporal features. Specifically, it was suggested that P2, N2 and P3 components were sensitive to self-referential processing (Chen et al., 2013; Tacikowski & Nowicka, 2010; Zhao et al., 2009) and we hypothesized that individual and collective self-referential processing would elicit different neural responses in these components.

## Materials and Methods

### Materials and participants

A total of 18 undergraduate students (10 females, 8 males) aged 18–25 years (mean age = 22.67, SD = 1.75) were invited to attend the electrophysiological study. All participants were right-handed and had normal or corrected-to-normal vision. In addition, the experimental protocol was approved by Ethics Committee of Hunan Normal University (2017115). Written informed consent was obtained from all the participants prior to the study and payment was given after experiment.

In addition, names of the participants, a famous person’s name, the names “Chinese” and “American” were selected as referential stimuli. The names of participants were presented visually as two-character or three-character Chinese words with the length matched between these name-categories. Moreover, 60 personality-trait adjectives were selected from established personality-trait adjective pools (Huang, 1992). Half of the trait-adjectives were positive and the other half were negative.

### Experimental task and procedure

**Figure 1:**
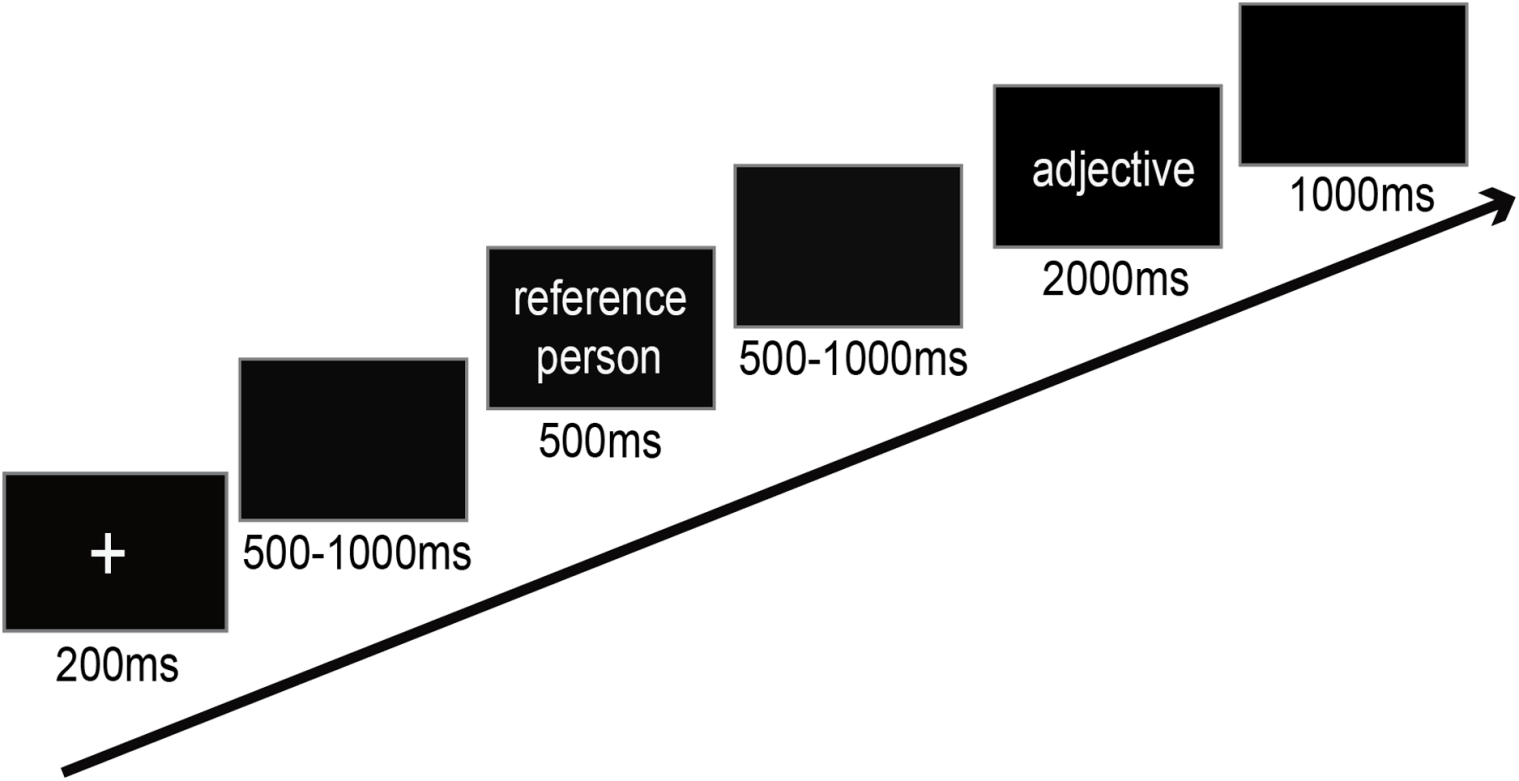
Schema of the design of the self-reference task.

Participants seated in a soundproof ERP laboratory at a distance of 120 cm from the screen center. There were four conditions during the experiment. Participants were asked to indicate whether the adjective described themselves (individual self-referential condition), a famous person (individual non-self-referential condition), Chinese (collective self-referential condition) or American (collective non-self-referential condition). For each trial, a fixation point appeared for 200 ms followed by a blank screen, the duration of which randomly varied from 500 ms to 1,000 ms. Subsequently, the reference person (self, a famous person, “Chinese” or “American”) was presented for 500 ms. After a 500–1,000 ms interval, a positive or negative trait adjective was presented for 2,000 ms (see Fig. 1). If a famous person’s name was presented just now, then participants were required to judge whether or not the adjective was appropriate to describe the famous person and press the appropriate keys (yes or no) to indicate their responses. The “yes” and “no” responses were counterbalanced across participants. The formal experiment totally consisted of six blocks and 240 trials. In addition, these four conditions were presented randomly and the adjectives were randomized across conditions in each block.

### Electrophysiological data recording and analysis

The electroencephalography (EEG) was recorded from 64 scalp electrodes mounted on an elastic cap (Brain Products) according to the extended International 10–20 system using FCz electrode as online reference. The impedance of each electrode was manipulated less than 10 kΩ. The offline electrophysiological data was first re-referenced to the average of the left and right mastoid electrode. After independent component analysis, separate EEG data was epoched from 200 ms before onset of the stimulus to 800 ms after its onset and baseline-corrected according to the mean voltage of a 200 ms pre-stimulus interval. Trials with amplitudes exceeding a threshold of ±80 µV during the recording epoch were eliminated from the final averages. Artifact-free ERP trials were averaged separately for each experimental condition.

Based on previous studies and the observation on the grand averaged waveforms, we analyzed three components, P2 (160–220 ms), N2 (270–330 ms), P3 (350–600 ms) with following regions: frontal (F3, F1, Fz, F2, F4), fronto-central (FC3, FC1, FCz, FC2, FC4), central (C3, C1, Cz, C2, C4), centro-parietal (CP3, CP1, CPz, CP2, CP4), parietal (P3, P1, Pz, P2, P4) regions. A three-way repeated analysis of variance (ANOVA) was conducted on the mean amplitudes of P2, N2 and P3 average amplitudes at each 50 ms of the 350–600 ms interval, with self-relevance (self vs. other), concept level (individual vs. collective) and brain regions (5 levels: frontal, fronto-central, central, centro-parietal and parietal) as within-subjects factors.

## Results

### Behavioral data

The repeated-measures ANOVA for the mean RTs showed significant main effects of concept level (*F* (1, 17) = 39.642, *P* < 0.001, η^2^_*P*_ = 0.7), self-relevance (*F* (1, 17) = 11.338, *P* = 0.004, η^2^_*P*_ = 0.4) and a marginally significant interaction between concept level and self-relevance (*F* (1, 17) = 4.158, *P* = 0.057, η^2^_*P*_ = 0.197). The reaction times for both individual (754.461 ms, *P* = 0.059) and collective (797.769 ms, *P* < 0.001) self-referential processing were shorter than for individual (793.496 ms) and collective (864.269 ms) non-self-referential processing, showing obvious individual and collective self-reference effect. Moreover, this effect was more prominent for individual than for collective self-referential processing.

### Electrophysiological data

#### P2 (160–220 ms)

**Figure 2:**
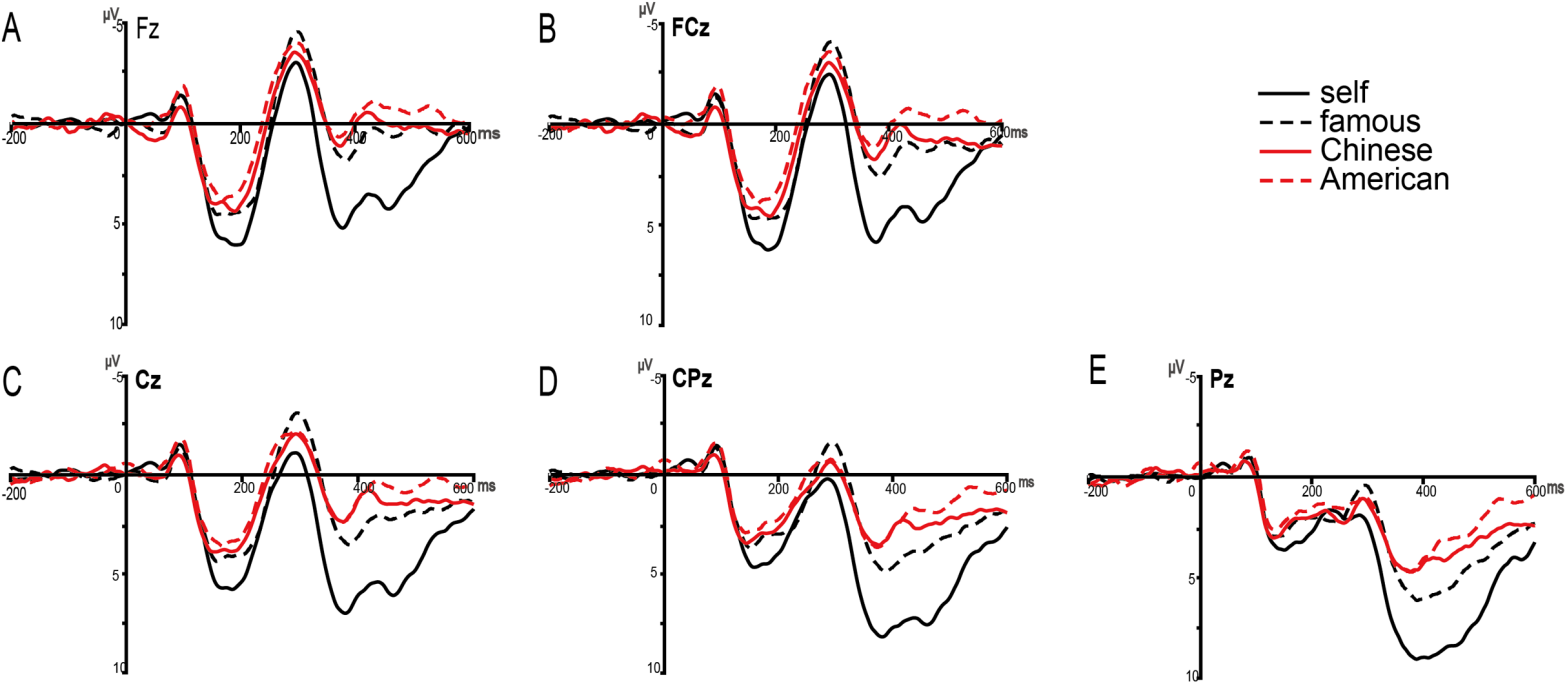
Image of grand average ERPs. Grand-average ERPs during self- (black solid lines), a famous person- (black dashed lines), Chinese- (red solid lines) and American-judgements (red dashed lines) at Fz, FCz, Cz, CPz and Pz electrode sites (A–E).

The ANOVA for P2 amplitudes showed significant interaction effect among self-relevance, concept level and regions (*F* (4, 68) = 3.939, *P* = 0.050, η^2^_*P*_ = 0.188). At the individual level, both the main effect of self-relevance (*F* (1, 17) = 6.193, *P* = 0.023, η^2^_*P*_ = 0.267) and the interaction effect between self-relevance and regions were significant (*F* (4, 68) = 6.160, *P* = 0.016, η^2^_*P*_ = 0.266). The simple effect analysis showed that larger P2 amplitudes were elicited in individual self-referential condition than in individual non-self-referential condition at frontal, fronto-central, central regions (*P*s < 0.05) but not at centro-parietal and parietal regions (*P*s > 0.05) (see Fig. 2). However, at the collective level, both the main effect of self-relevance and the interaction effect between self-relevance and regions were not significant (*P*s > 0.1).

#### N2 (270–330 ms)

The ANOVA for N2 amplitudes showed the main effects of self-relevance (*F* (1, 17) = 11.599, *P* = 0.003, η^2^_*P*_ = 0.406) and regions (*F* (4, 68) = 31.357, *P* < 0.001, η^2^_*P*_ = 0.648). In addition, the interaction effect between concept level and self-relevance was also significant (*F* (1, 17) = 5.454, *P* = 0.032, η^2^_*P*_ = 0.243). At the individual level, smaller N2 amplitudes were elicited in individual self-referential condition than in individual non-self-referential condition (*F* (1, 17) = 13.246, *P* = 0.002, η^2^_*P*_ = 0.438). However, at the collective level, the main effect of self-relevance was not significant (*F* (1, 17) = 1.318, *P* = 0.267, η^2^_*P*_ = 0.072).

#### P3 (350–600 ms)

##### 350–450 ms

The ANOVA for averaged amplitudes during 350–400 ms and 400–450 ms time intervals both showed significant main effects for self-relevance (*F*s (1, 17) > 15.725, *P*s = 0.001, η^2^_*P*_ > 0.481), concept level (*F*s (1, 17) > 32.767, *P*s < 0.001, η^2^_*P*_ > 0.658) and regions (*F*s (4, 68) > 28.082, *P*s < 0.001, η^2^_*P*_ > 0.623). In addition, the interaction effect between self-relevance and concept level was significant (*F*s (1, 17) > 10.229, *P*s < 0.005, η^2^_*P*_ > 0.376). At the individual level, larger P3 amplitudes were elicited in individual self-referential condition than in individual non-self-referential condition (*F*s (1, 17) > 21.171, *P*s < 0.001, η^2^_*P*_ > 0.555). However, at the collective level, no significant difference was observed between collective self-referential and collective non-self-referential processing (*F*s (1, 17) > 0.308, *P*s > 0.1, η^2^_*P*_ > 0.018) (see Fig. 2).

##### 450–500 ms

The ANOVA for P3 amplitudes during 450–500 ms showed significant main effects for self-relevance (*F* (1, 17) = 23.742, *P* < 0.001, η^2^_*P*_ = 0.583), concept level (*F* (1, 17) = 50.977, *P* < 0.001, η^2^_*P*_ = 0.750) and regions (*F* (4, 68) = 23.446, *P* < 0.001, η^2^_*P*_ = 0.580). Moreover, the interaction effect between self-relevance and concept level was also significant (*F* (1, 17) = 12.103, *P* = 0.003, η^2^_*P*_ = 0.416). The individual self-referential condition elicited larger P3 amplitudes than individual non-self-referential condition (*F* (1, 17) = 27.058, *P* < 0.001, η^2^_*P*_ = 0.614) (see Fig. 2). In addition, the collective self-referential condition also elicited larger P3 amplitudes than collective non-self-referential condition (*F* (1, 17) = 4.588, *P* = 0.047, η^2^_*P*_ = 0.213). The individual self-reference effect indicated by the differences between individual self-referential and individual non-self-referential conditions was larger than the collective self-reference effect indicated by the differences between collective self-referential and collective non-self-referential conditions (*P* < 0.01) (see Fig. 4).

##### 500–600 ms

The ANOVA for averaged amplitudes during 500–550 ms and 550–600 ms time intervals both showed significant main effect for self-relevance (*F*s (1, 17) > 4.438, *P*s < 0.05, η^2^_*P*_ > 0.207). However, no significant interaction was observed between self-relevance and concept level (*F*s (1, 17) > 0.057, *P*s > 0.1, η^2^_*P*_ > 0.003).

## Discussion

The present study examined the temporal features in individual and collective self-referential processing by using ERPs. The behavioral results showed both obvious individual and collective self-referential effect. Moreover, this effect was more prominent for individual than for collective self-referential processing, to some extent reflecting differences in processing of individual and collective self. Our ERP results showed that individual self-referential processing elicited larger P2 and smaller N2 amplitudes as compared to individual non-self-referential processing, whereas no significant differences were observed between collective self-referential and collective non-self-referential processing at these stages. In addition, both individual and collective self-referential processing elicited larger P3 amplitudes than individual and collective non-self-referential processing. However, the strength of individual self-reference effect, which is indicated by the P3 amplitude differences between individual self-referential and individual non-self-referential processing, was larger than that of the collective self-reference effect indicated by the P3 amplitude differences between collective self-referential and collective non-self-referential processing during 350–500 ms. Moreover, the strength of individual and collective self-reference effects was comparable during 500–600 ms. These findings demonstrated distinct temporal features in individual and collective self-referential processing.

It was suggested that P2 component was related to allocation of attentional resources (Carretié et al., 2011) and larger P2 amplitudes indicated increased attention recruitment (Carretié et al., 2004; Chen et al., 2013). More specifically, highly salient stimuli such as negative emotional stimuli usually elicited larger P2 amplitudes than positive or neutral emotional stimuli (Carretié et al., 2001, 2004). It has been also found that self-name elicited larger P2 amplitudes than other names (Chen, Shui & Zhong, 2015; Chen et al., 2011). Consistent with previous studies, the present study also observed larger P2 amplitudes in self-condition (individual self) compared with the famous person-condition. However, no significant P2 differences were observed between “Chinese” (collective self) and “American” conditions. These results demonstrated an obvious individual self-reference effect at the early P2 stage whereas no collective self-reference effect was observed at this stage. This might be because collective self-relevant stimuli were not as salient and emotionally significant as individual self-relevant stimuli. A previous ERP study demonstrated the degree effect of self-relevance, and found that the P2 component was only sensitive to the high self-relevant stimulus rather than moderate and low self-relevant stimuli (Chen et al., 2011). Thus, individual rather than collective self-relevant stimuli might be more self-relevant to participants. These findings were also consistent with the individual-self primacy hypothesis, which suggested that the individual-self was motivationally primary and closer to the core of self-definition (Gaertner et al., 2002, 2008, 2012; Sedikides et al., 2013).

The N2 component was observed in four experimental conditions during the 270–330 ms time interval. It is a processing stage between automatic and controlled phases (Näätänen & Picton, 1986). Similar to the P2 effect observed above, the present study also observed an obvious individual self-reference effect at the N2 processing stage, and smaller N2 amplitudes were elicited by individual self-referential processing than by individual non-self-referential processing. This was consistent with previous studies showing that subjects’ own handwritings and names elicited smaller N2 amplitudes than other handwritings and names (Chen et al., 2008, 2011). However, no collective self-reference effect was observed at this stage.

More importantly, both individual and collective self-reference effects occurred at later P3 processing stage, and larger P3 amplitudes were elicited by individual and collective self-referential processing than by individual and collective non-self-referential processing. It should be noted that collective self-reference effect didn’t occur until the P3 stage. Different from the earlier P2 and N2 components, the P3 component was considered to be related to late controlled processes and could be modulated by top-down attention resources (Yuan et al., 2007, 2008). In addition, the P3 amplitude was related to self-relevant stimuli and larger P3 amplitudes were elicited by self-relevant stimuli than by non-self-relevant stimuli (Chen et al., 2011; Fan et al., 2013; Zhao et al., 2009). With more controlled processing resources, both individual and collective self-referential processing, due to their self-relevance, could be distinguished from non-self-referential processing at this elaborate processing stage.

**Figure 3:**
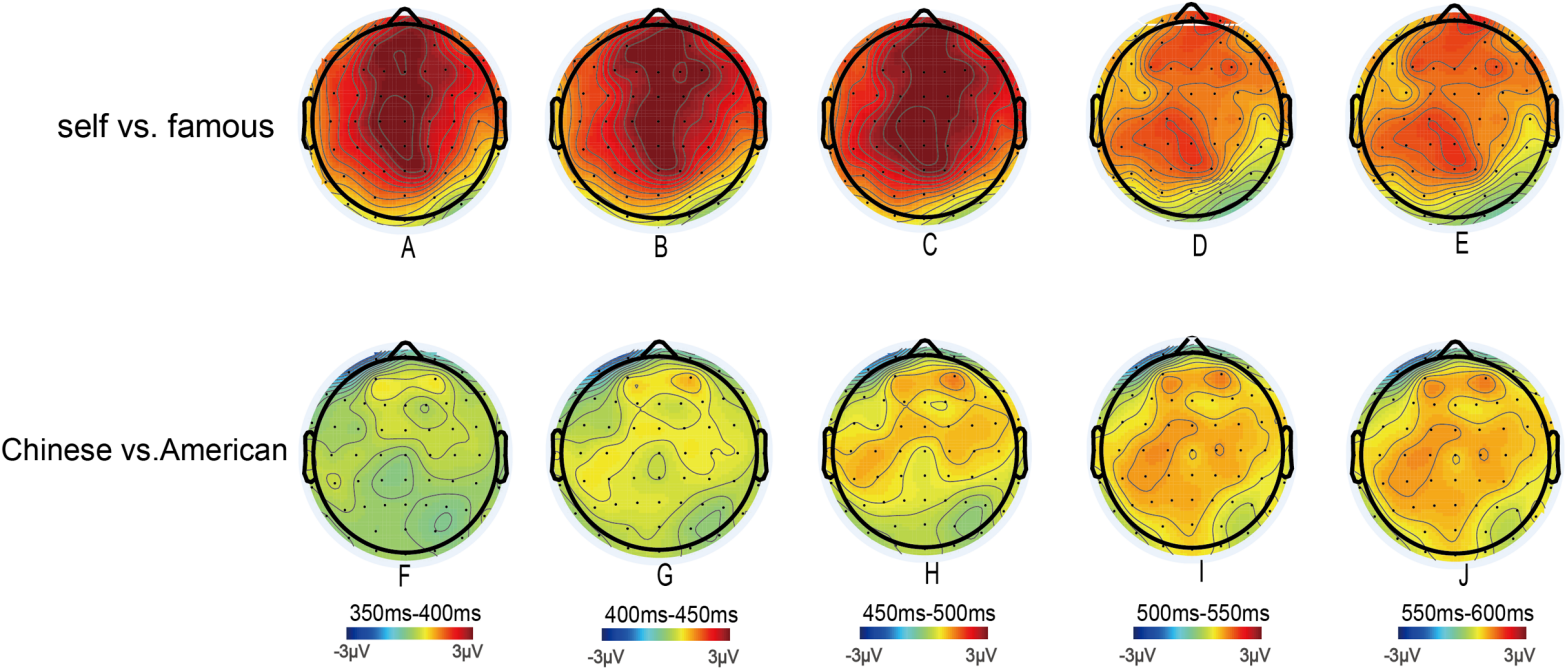
Image of topographical maps. Topographical maps of the voltage amplitudes for self-name-famous person’s name (A–E) and the name “Chinese”–“American” (F–J) difference ERPs in the 350–400 ms, 400–450 ms, 450–500 ms, 500–550 ms and 550–600 ms intervals.

**Figure 4:**
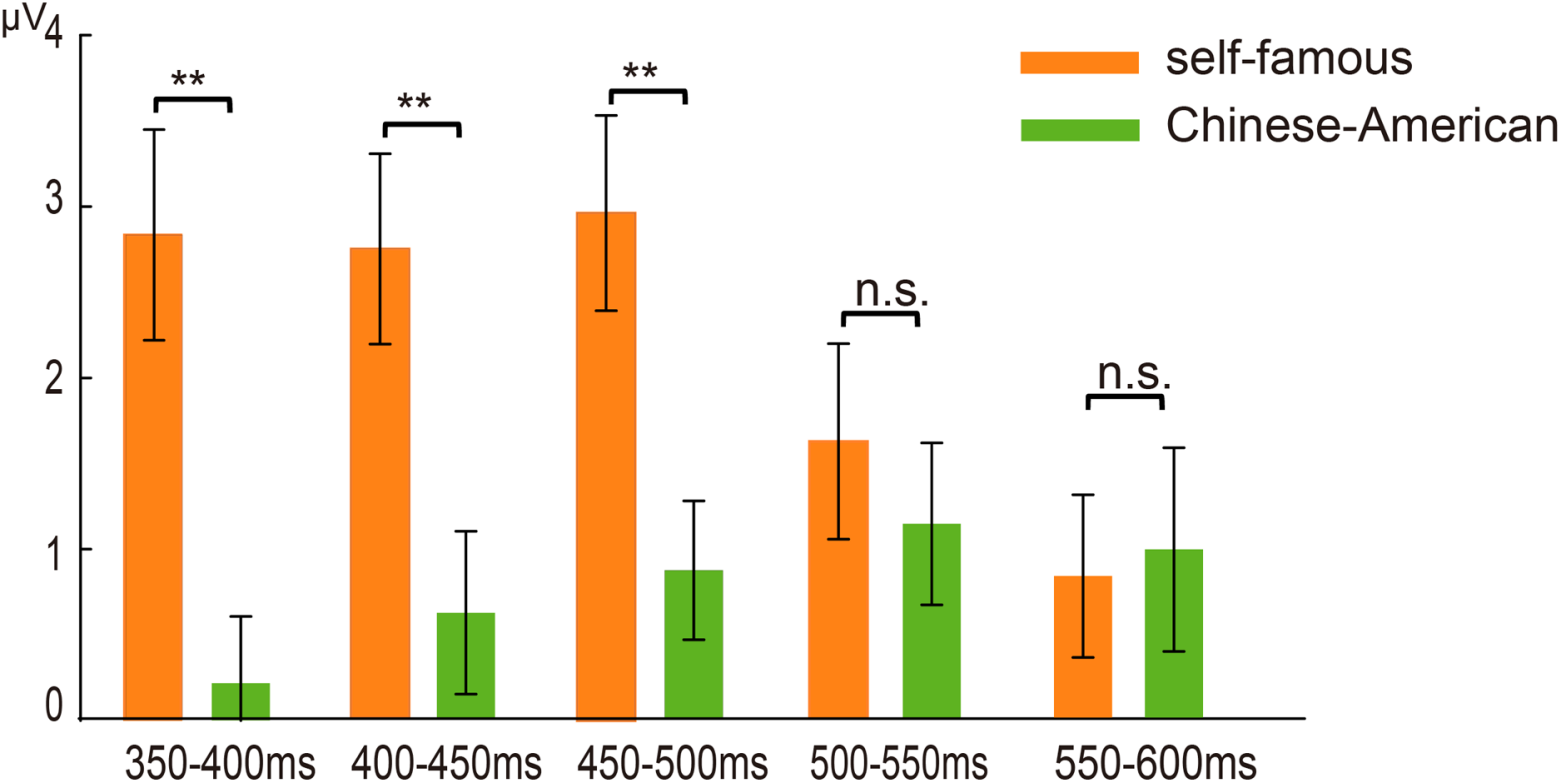
Individual and collective self-reference effect. Schematic illustration of the individual self-reference effect (self-famous name amplitude differences) and collective self-reference effect (the names “Chinese”–“American” amplitude differences) in the 350–400 ms, 400–450 ms, 450–500 ms, 500–550 ms and 550–600 ms intervals. **P < 0.01.

Although both individual and collective self-reference effects were observed at P3 stage, these effects were different among different time windows of P3 stage (see Fig. 3). The collective self-reference effect was occurring during 450–600 ms (see Fig. 3). In addition, the individual self-reference effect was more prominent than collective self-reference effect during 350–500 ms, whereas no significant difference was observed between individual and collective self-reference effect in the 500–600 ms interval (see Fig. 4). To some extent, these findings might indicated that there were different neurocognitive mechanisms underlying the individual and collective self-referential processing at different time windows of P3 stage. Although the cognitive and neural mechanisms of self-referential processing have been studied extensively (Gusnard et al., 2001; Mu & Han, 2010; Tacikowski, Brechmann & Nowicka, 2013; Tamura, Mizuba & Iramina, 2016; Woźniak, Kourtis & Knoblich, 2018), limited studies directly compared different components or aspects of the self (Hu et al., 2016; Kircher et al., 2000; Lou et al., 2004). For example, Hu et al. (2016) compared the neural representations of physical and psychological self using ALE meta-analysis and found both distinct and common neural substrates during physical and psychological self-representation. Thus, it would be necessary and interesting to directly compare the neural representations of individual and collective self by using high-spatial-resolution functional magnetic resonance imaging (fMRI) in the future.

## Conclusions

In conclusion, in addition to previous studies emphasizing on a single aspect of the self, the current study revealed diﬀerent temporal dynamics of individual and collective self-referential processing, which increased our understanding of self-concept. The individual self-reference effect appeared not only at the early P2 and N2 stages, but also at the late P3 stage, whereas the collective self-reference effect did not appear until the late P3 stage. Moreover, the individual self-reference effect was more prominent than collective self-reference effect in the 350–500 ms interval, whereas the individual and collective self-reference effects were comparable in the 500–600 ms time window.

## Supplemental Information

10.7717/peerj.8917/supp-1 Supplemental Information 1 Behavioral data.

10.7717/peerj.8917/supp-2 Supplemental Information 2 The ERP data (raw data) of individual and collective self-referential processing.

## Additional Information and Declarations

### Data Availability

The following information was supplied regarding data availability: The raw data are available in the Supplemental Files.
